# Crystal Structure, Cation Occupation, and Phase Transitions in Ba_4_(Li_*x*_Na_1–*x*_)_2_Nb_10_O_30_ Tetragonal Tungsten Bronzes

**DOI:** 10.1021/acs.inorgchem.4c04461

**Published:** 2025-01-03

**Authors:** Nora Statle Løndal, Benjamin A. D. Williamson, Ola G. Grendal, Julian Walker, Mari-Ann Einarsrud, Tor Grande

**Affiliations:** Department of Material Science and Engineering, NTNU Norwegian University of Science and Technology, Trondheim 7491, Norway

## Abstract

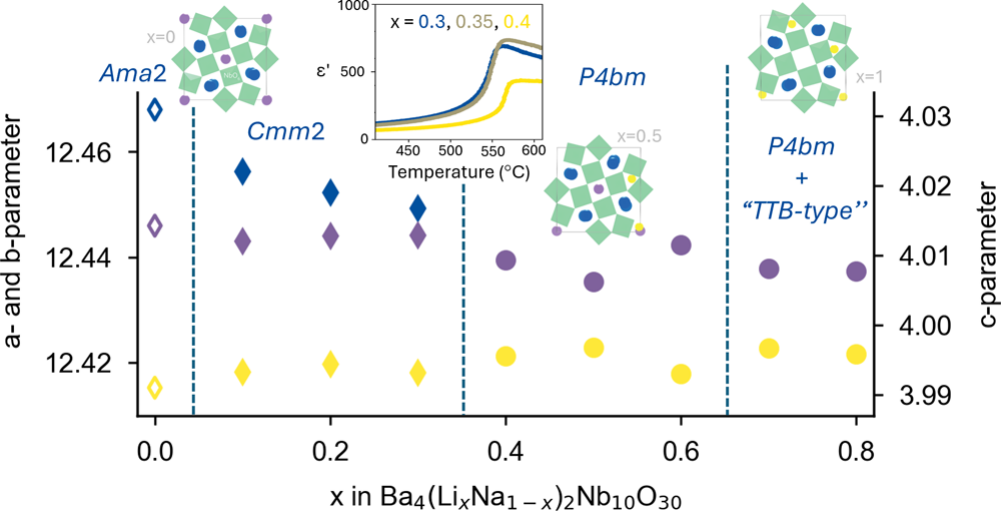

The chemical flexibility of the tetragonal tungsten bronze (TTB) structure offers a large potential for compositional engineering. Cation size and vacancy concentration are known to affect its structure, cation disorder, and functional properties. However, the compositional complexity also makes the TTB structure challenging to understand. Here, we investigate the solid solution between orthorhombic Ba_4_Na_2_Nb_10_O_30_ and tetragonal Ba_4_Li_2_Nb_10_O_30_ TTBs. Ceramics of the composition Ba_4_(Li_*x*_Na_1–*x*_)_2_Nb_5_O_30_ were achieved through solid-state synthesis. The crystal structures were investigated by powder X-ray diffraction, which revealed a transition from orthorhombic (*Cmm*2) to tetragonal (*P*4*bm*) symmetry for Li content, *x*, between 0.3 and 0.35. For materials of compositions close to this transition, a *T*_C_ of 557–572 °C was observed. Site occupancies of Li-, Na-, and Ba-ions were investigated through Rietveld refinement, complemented by DFT calculations, and were discussed with respect to the stability of the TTB structure. While the Na configuration on the A1- and A2-sites depends on the Na concentration, the Ba configuration appears constant over the compositional range. The Li-ions solely occupy C-sites, and with increasing Li contents, higher vacancy concentrations are created on the A-sites. For compositions of *x* > 0.60, a second phase, with a TTB-related structure, appears. This work has increased the understanding of the composition–structure relationship in TTBs.

## Abstract 2

Here, we investigate tetragonal tungsten bronzes (TTBs) of the composition Ba_4_(Li_*x*_Na_1−*x*_)_2_Nb_5_O_30_ (BLNN*x*). The crystal structures were investigated by powder X-ray diffraction, which revealed a transition from orthorhombic to tetragonal symmetry for Li content, *x*, between 0.30 and 0.35. Dielectric properties, which were characterized in the vicinity of the phase boundary, indicate ferroelectric transitions in the range of 557−572 °C. The Li-, Na-, and Ba-ion site occupancies are discussed with respect to the structure and stability of the TTB structure.

## Introduction

Ferroelectrics possess high dielectric constants and piezoelectric coefficients and are therefore key elements of capacitors, sensors, and actuators, all of which play an important role in modern electronic devices.^1,2^ PbZr_1–*x*_Ti_*x*_O_3_ (PZT) is the most versatile and widely used ferroelectric material. However, due to the toxicity of lead and its ban from electronic appliances under the restriction of hazardous compounds (RoHS) directive^3−5^ by the European Union, a search for alternative lead-free ferroelectrics is ongoing.

The second largest family of oxide ferroelectrics, after perovskites, is tetragonal tungsten bronze (TTB). The general formula of TTB is A2_4_A1_2_C_4_B1_2_B2_8_O_30_, and the structure consists of a network of corner-sharing BO_6_ octahedra, which construct four pentagonal, two square, and four trigonal channels that run along the *c*-axis of the structure and make the A2-, A1- and C-sites, respectively.^6^ The large flexibility of the structure allows for broader compositional engineering compared with perovskites. TTBs are also prone to high Curie temperatures (*T*_C_), which are needed for applications in, e.g., aerospace and automotive industries^6,7^ It is known that factors such as cation size, valency, and vacancy concentration affect the structure, cation disorder, functional properties, such as the *T*_C_, as well as the development of relaxor properties.^8,9^ However, the TTB framework comes with a level of complexity that is, compared to perovskites, far less studied and in no way fully understood.

The wide success of PZT has often been assigned to the system having a so-called morphotropic phase boundary (MPB), which is a compositionally induced and close-to-temperature-independent phase boundary between two structurally distinct phases.^10,11^ In the search for lead-free material systems that can replace PZT, MPB systems are sought after. TTBs are known to form MPB systems.^12^ Some lead-free TTB systems reported with MPBs are (Ba_1–*x*_Sr_*x*_)_4_Na_2_Nb_10_O_30_,^13,14^ Ba_4–*x*_Sr_*x*_K_2–*y*_Na_*y*_Nb_10_O_30_^15,16^, and Ba_6–*x*_Sr_*x*_Ti_2_Nb_8_O_30_,^17^ and they typically show enhanced properties across a tetragonal-to-orthorhombic phase transition. We have also recently reported enhanced properties over two proximate phase transitions in solid solutions of Ba_4_Na_2_Nb_10_O_30_ (BNN) and K_4_Bi_2_Nb_10_O_30_.^18^

The two TTBs BNN^19^ and Ba_4_Li_2_Nb_10_O_30_ (BLN)^20^ both have high *T*_C_ values of 570 and 600 °C, as well as orthorhombic and tetragonal room-temperature symmetries, respectively. Thus, the two TTBs make good candidates for end members of a lead-free solid solution with MPB property enhancements eligible for high-temperature applications. Additionally, as Na- and Li-ions are reported to have site preference for A1- and C-sites, respectively, the BLNN*x* (0 ≤ *x* ≤ 1) system is a good candidate to increase our understanding of how the structure, properties, and cation site occupation in TTBs are affected by cation size and vacancy concentration. Finally, as pure BLN is reported to need excess Ba to form a stable TTB, the BLNN system offers an opportunity to explore a stability limit within the TTB framework.

Literature on the BLNN*x* system is, to our knowledge, limited to the report by Masuda and Wada from 1975^21^ on the single crystal BLNN*x* with *x* values of 0.2 and 0.4, where orthorhombic symmetry, a *T*_C_ of 490 °C, and hysteresis loops at 25 and 105 °C were reported for the compound with an *x* of 0.2. In the present work, we investigate the whole compositional range between orthorhombic BNN and tetragonal BLN with respect to the crystallographic site occupation of the Li-, Na- and Ba-ions in the TTB structure, any observed compositionally induced phase transitions with an interest in exploring it as an MPB system, and stability limits within the TTB framework. Structure and cation site occupation are assessed by the Pawley and Rietveld refinement of both high-resolution synchrotron and lab powder X-ray diffraction patterns complemented by DFT calculations. Phase transition temperatures were identified with temperature-dependent dielectric permittivity measurements.

## Experimental Section

### Solid-State Synthesis

Ceramic solid solutions of BNN and BLN were synthesized through a two-step solid-state synthesis route.^18,22,23^ Solid solutions, BLNN*x*, with *x* = 0.10, 0.20, 0.30, 0.35, 0.40, 0.50, 0.60, 0.70, 0.80, 0.85, 0.90, 0.95, and 1.0 were prepared. In the first step, Nb_2_O_5_ was separately mixed with Li_2_CO_3_, Na_2_CO_3_, and BaCO_3_ to prepare the niobate precursor compounds LiNbO_3_, NaNbO_3_, and BaNb_2_O_6_, respectively. The chemicals were dried at 200 °C prior to weighing out stoichiometric amounts followed by mixing in ethanol by ball milling for 2 h. The ethanol was evaporated from the powder mixtures before uniaxially pressing pellets with a diameter of 15–20 mm, depending on the precursor, at ∼40 MPa. The pellets of LiNbO_3_ were prepared by calcination at 750 °C for 4 h in alumina crucibles with a lid. NaNbO_3_ pellets were calcinated at 700 °C for 4 h and then further at 900 °C for 12 h. BaNb_2_O_6_ was achieved by calcination at 1100 °C for 6 h. The precursor pellets were crushed and ground into fine powder in a boron carbide mortar and dried at 120 °C. Powders of BaNb_2_O_6_, NaNbO_3_, and LiNbO_3_ were mixed in stoichiometric amounts in ethanol by ball milling for approximately 24 h. Ethanol was evaporated from the mixtures before 10 mm-diameter pellets were uniaxially pressed at ∼90 MPa and sintered in alumina crucibles with a lid. To avoid loss of alkali during sintering, the pellets were covered by a sacrificial powder of BLNN50 (*x* = 0.50). All solid solutions were sintered at 1285 °C for 6 h at a heating rate of 200 °C/h. Powders of the sintered BLNN*x* ceramics were prepared by crushing and grinding in a boron carbide mortar.

### Structural Characterization

Initially, XRD patterns for all BLNN solid solutions were recorded with a divergence slit of 0.1°, a step size of 0.013° from 5 to 75 2θ for an acquisition time of 1 h, and a step size of 0.014° from 5 to 110 2θ for an acquisition time of 10 h. Both types of measurements were done using a Bruker DaVinci1 diffractometer with Cu Ka radiation equipped with a LynxEye Super Speed detector under ambient conditions. For the BLNN*x* materials with compositions *x* = 0.10, 0.20, 0.30, 0.40, 0.50, 0.60, 0.70, and 0.80, high-resolution XRD data were collected in transmission geometry at the ID22 beamline at the European Synchrotron Radiation Facility (ESRF).^24,25^ The powders were transferred into 0.5 mm-diameter borosilicate capillaries, and powder diffraction patterns were collected at ambient conditions up to a *Q* of about 10 Å^–1^, using a wavelength of λ = 0.3543 Å. The wavelength was calibrated using a silicon standard (NIST, 640c).

Analysis of the collected patterns was done using the Bruker AXS software packages Diffrac.Eva 5.2 and Topas 5. Pawley refinements were used to determine the space group symmetry of each composition prior to Rietveld refinement. Nominal stoichiometry was assumed for all compositions during the Rietveld refinement. The peak shape was described using the fundamental parameter model.^26^ Lattice parameters and sample displacement were refined, and Chebyshev polynomials were used for the background. All diffractograms of the solid solutions were first refined by the tetragonal *P*4*bm* space group, but some BNN-rich materials show orthorhombic splitting. For the materials showing orthorhombic splitting, the orthorhombic space groups *Cmm*2, *Ama*2, and *Pba*2 were tested. For pure BLN (*x* = 1), additional Pawley refinement using the *Amm*2 space group was carried out, based on a match in the Crystallography Open Database for an observed secondary phase.

Rietveld refinement was carried out on HR-XRD patterns with either the *P*4*bm* or *Cmm*2 space group, depending on whether orthorhombic splitting was observed or not. In each refinement, a background function by Chebyshev polynomials, zero error, scale factor, a Simple_Axial_Model asymmetry parameter, a Voigt function for the instrumental peak shape, lattice parameters, atomic site parameters (11 for *Cmm*2 and 4 for *P*4*bm*), and two site occupation parameters for A1-, A2-, and C-sites were refined. The A1-, A2-, and C-site occupancy was refined according to constraints summarized in Table 1. Atomic displacement parameters (*B*_eq_) from the combined X-ray and neutron diffraction studies by Olsen et al.^27^ were used and kept constant throughout the refinements. It was assumed that Ba and Na can occupy the A1- and A2-sites, while Li can occupy the A1- and C-sites. This opens up the possibility of site vacancies to be present at all three sites. Free refinement of occupation on A1-, A2-, and C-sites was initially tested, but a correlation between the free parameters occurred. Thus, for each BLNN*x* composition (*x* = 0.10, 0.20, 0.30, 0.40, 0.50, 0.60, 0.70, 0.80), the total occupation on the A2-site (Tot_A2_) was manually adjusted between 1 and a minimum value governed by the Li/Na content when an occupation of 1 was achieved at the A1-site, and a refinement was carried out at each defined configuration. A refinement of an ordered cation configuration, with all Ba, Na, and Li fixed to occupy the A2-, A1-, and C-sites, respectively, was also carried out for each composition.

**Table 1:** Constraints and Limits Applied for Cation Site Occupancy upon Rietveld Refinement of the BLNN HR-XRD Patterns

Site	cation	constraint	min limit	max limit
A1	Ba	Ba_A1_ = 2(1–Tot_A2_) + Na_Tot_–Na_A1_		
	Na	**Na**_**A1**_	0	Na_Tot_
	Li	**Li**_**A1**_	0	1–Na_A1_–Ba_A1_
A2	Ba	Tot_A2_–(Na_Tot_–Na_A1_)/2		
	Na	(Na_Tot_–Na_A1_)/2		
C	Li	(Li_Tot_–Li_A1_)/2		

The parameters **Na**_**A1**_ and **Li**_**A1**_ are refined freely and give the occupancy of Na and Li on the A1-site, respectively. The constants Na_Tot_ and Li_Tot_ denote the total Na and Li contents, respectively, in the specific BLNN solid solution composition refined. The constant Tot_A2_ denotes the total occupation of cations on the A2-site, which is set manually in each specific refinement.

### Dielectric Measurements

Ceramics for permittivity measurements were additionally cold-isostatically pressed at ∼170 MPa prior to sintering. The density of the samples was determined with the caliper method, and the theoretical density was calculated based on the cell volume, given by the lattice parameters, and molar mass of each stoichiometric compound. Approximately 100 μm of the surface from the as-sintered pellets was ground away with SiC paper, and electrodes were prepared using a platinum paste, which was baked at 900 °C for 10 min. Dielectric permittivity measurements were carried out with a Novocontrol Alpha-A HVB 4000 impedance analyzer with a Norecs ProboStat A-5 sample cell in a tubular furnace connected to a Eurotherm 2408 temperature controller. Data were collected through the Novocontrol WinDETA software at frequencies of 100 Hz, 1 kHz, and 10 kHz continuously while heating and cooling between 30 and 700 °C with a heating/cooling rate of 2 °C/min for two full cycles. Data from heating during the second cycle is reported.

## Computational Section

The Vienna ab initio simulation package^28−31^ was used to perform density functional theory (DFT) calculations on BLNN*x*. In order to describe the interactions between the core and valence electrons (Ba[Xe], Li(full), Na[He], Nb[Kr], O[He]), the projector augmented wave^32^ method was used in addition to the Perdew–Burke–Ernzerhof (PBEsol) functional^33,34^ to describe the exchange and correlation. PBEsol consistently calculates good descriptions of the structural properties of condensed matter phases relative to the experiment and has been used to great success in terms of ionic disorder and alloying,^35,36^ as well as in previous works on TTB compositions.^23,27,37,38^

Solid solutions and disordered cells were generated using the bsym python package^39^ with analysis of the Gibbs free energy of mixing Δ*G*_mix_ and disorder calculated in line with a previous work^23^ based on formalism outlined by Grau-Crespo et al.,^40^ which can also be found in the Supporting Information (SI). The disordered cells were initialized using the well-defined BNN structure in the *P*4/*mbm* aristotype space group. Solid solutions between BNN and BLN were performed in addition to site disorder between the A1- and A2-sites, as well as A1- and C-sites within the structures. Configurational degeneracies are provided in SI Tables S9 and S10 for A1/A2 and A1/C-site disorders, respectively. All structures were then subjected to full geometry relaxation (ionic positions, lattice parameters, and volume) using a Γ-centered k-point grid of 2 × 2 × 6 and a plane-wave energy cutoff of 500 eV toward a force convergence criterion of <0.01 eV Å^–1^. Probabilistically averaged structural properties are provided in SI Tables S13 and S14.

## Results

### Structural Analysis

**Figure 1:**
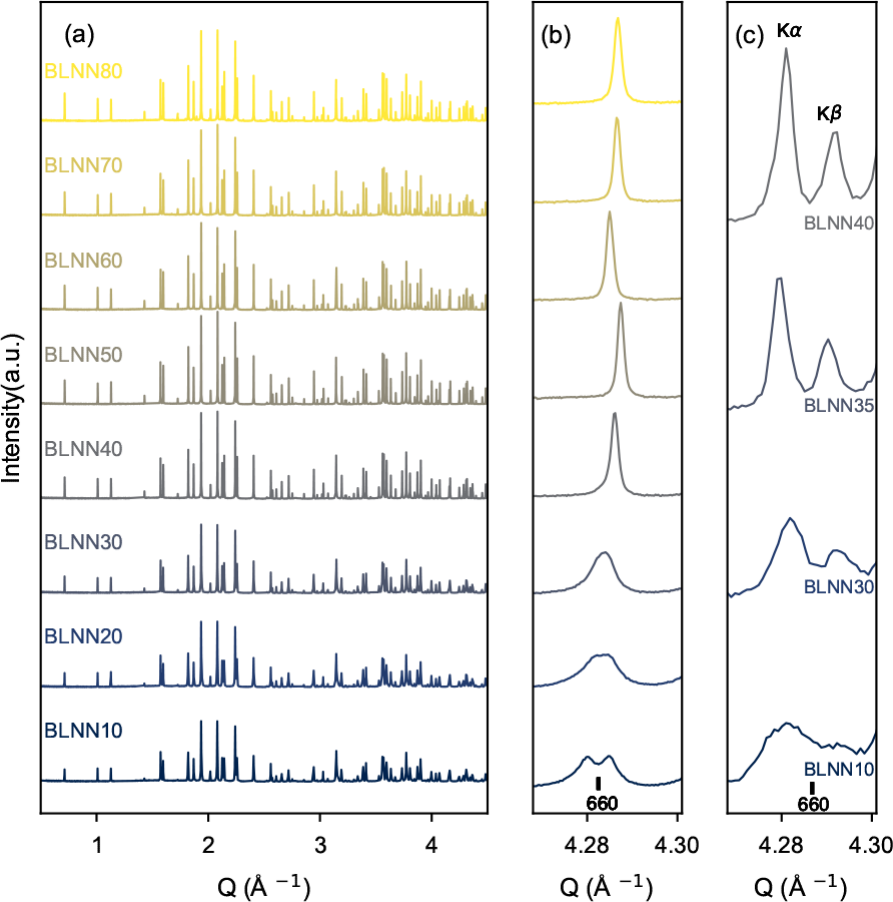
XRD patterns of BLNN solid solutions from a synchrotron X-ray source (HR-XRD) and a copper filament source (F-XRD). The HR-XRD pattern is shown from (a) 0.5 to 4.5 Å^–1^ as well as in a narrower section (b) focusing on the 660-reflection (with respect to the TTB aristotype *P*4/*mbm* symmetry). The 660-reflection is (c) displayed for selected F-XRD patterns.

Phase-pure TTB-structured compounds were achieved for BLNN compositions with a Li content of up to 0.6. Only minor traces of a secondary phase were observed for *x* values of 0.70 and 0.80, while the secondary phase, which is a “TTB-type” structure, became more prominent with a higher Li content. Figure 1 displays the XRD patterns, from a (a, b) synchrotron X-ray source (HR-XRD) and (c) copper filament source (F-XRD) for the BLNN*x* compositions in the range 0.10 ≤ *x* ≤ 0.80 (BLNN10-BLNN80).

In the HR-XRD patterns (Figure 1b) for BLNN10 (*x* = 0.10), the 660-reflection is clearly split, while the splitting decreases with increasing Li content to BLNN30. For BLNN40, a sharp single reflection, indicating that a transition into a higher symmetry takes place, is exhibited. The F-XRD patterns for compositions with Li contents of 0.10, 0.30, 0.35, and 0.40 (Figure 1c) have a lower resolution than the HR-XRD, as well as both *K*_α_ and *K*_β_ reflections. This makes it more challenging to observe where the splitting of the 660-reflection is present and not; however, there is clearly a narrowing of the peaks evolving from BLNN10 to BLNN30 before sharpening further at BLNN35. No distinct narrowing is seen between BLNN35 and BLNN40, suggesting that the transition takes place between *x* = 0.3 and 0.35.

**Figure 2:**
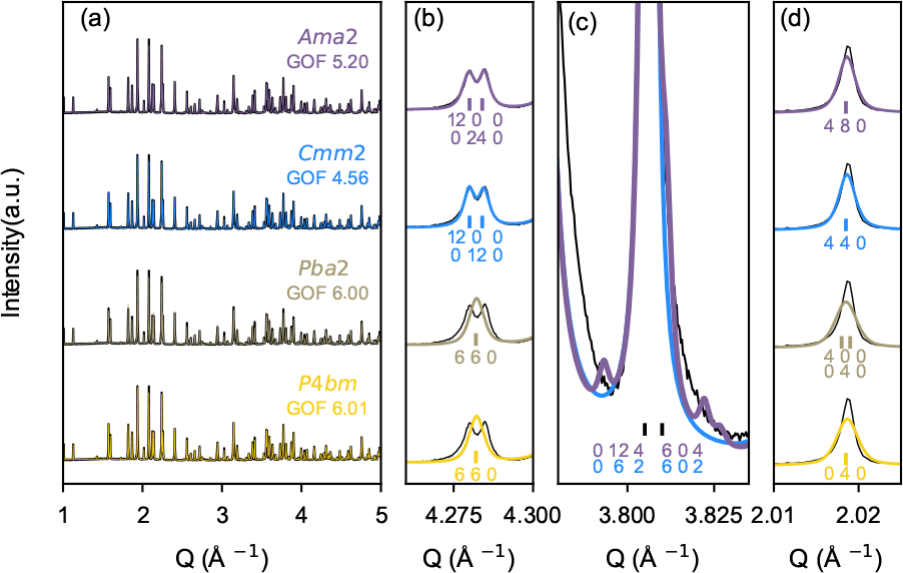
Pawley refinement of the HR-XRD pattern of BLNN10 (*x* = 0.10) with the four space groups *P*4*bm*, *Pba*2, *Cmm*2, and *Ama*2 at (a) 1–5 Å^–1^, (b) 4.26–4.30 Å^–1^, (c) 3.78–3.83 Å^–1^, and (d) 2.010–2.025 Å^–1^. In (a), (b), and (c), selected reflections are indexed according to the respective space groups.

Pawley refinement of HR-XRD data of the BLNN solid solution compounds indicates a tetragonal *P*4*bm* symmetry for compositions with Li contents of 0.4 and higher, while for lower Li contents, an orthorhombic structure is suggested. The orthorhombic symmetry is clearly observed from the splitting of certain high-angle (hk0) reflections (see the (660) reflection in Figure 1), which is not allowed by a tetragonal unit cell (see Figures 2b and S1 (SI)), previously also demonstrated for other TTB systems.^18,41^

Pawley refinement with the three different orthorhombic space groups *Pba*2, *Cmm*2, and *Ama*2, as seen for BLNN10 (*x* = 0.10) in Figure 2, shows that *Cmm*2 is the space group that best describes the solid solutions with Li contents from 0.10 to 0.30, at ambient temperature. The *Ama*2 space group gives a relatively good fit; however, some small additional reflections (Figure 2c) were generated in the fit, but not observed in the data, suggesting overfitting of the data compared to the *Cmm*2 symmetry, which is reflected in the better quality of fit (low GOF values) for *Cmm*2 than for *Ama*2. The orthorhombic *Pba*2 space group gives splitting of other reflections, such as the 440 (aristotype) as seen in Figure 2d, than what is observed for *Ama*2 and *Cmm*2. A section of the Pawley refinement with *P*4*bm* and *Cmm*2 symmetries for compositions with Li contents from 0.10 to 0.40 is displayed in SI Figure S2.

The secondary phase observed in materials with a high Li content (SI Figure S2a) was identified to be isostructural to the structure of Ba_4_MgTa_10_O_30_ (BMT),^42^ which has an *Amm*2 symmetry. This can be seen for pure BLN (*x* = 1) in SI Figure S2b. The “TTB-type” structure resembles the TTB structure, built from the same block of octahedra (SI Figure S3); however, the octahedra are differently organized so that the pentagonal, quadrilateral, and trigonal sites are coordinated differently. The quadrilateral “A1-site” in the “TTB-type” structure is deltoidal and thus also smaller compared to the square A1-site of the TTB structure. This could indicate that decreasing occupation on the A1-site in the TTB structure leads to a collapse of the TTB structure, making the “TTB-type” structure more favorable due to its smaller “A1-site”. The Mg cations are reported on the “A1-site” in BMT, and thus, we expect the same for the slightly larger Li cations of BLNN.

**Figure 3:**
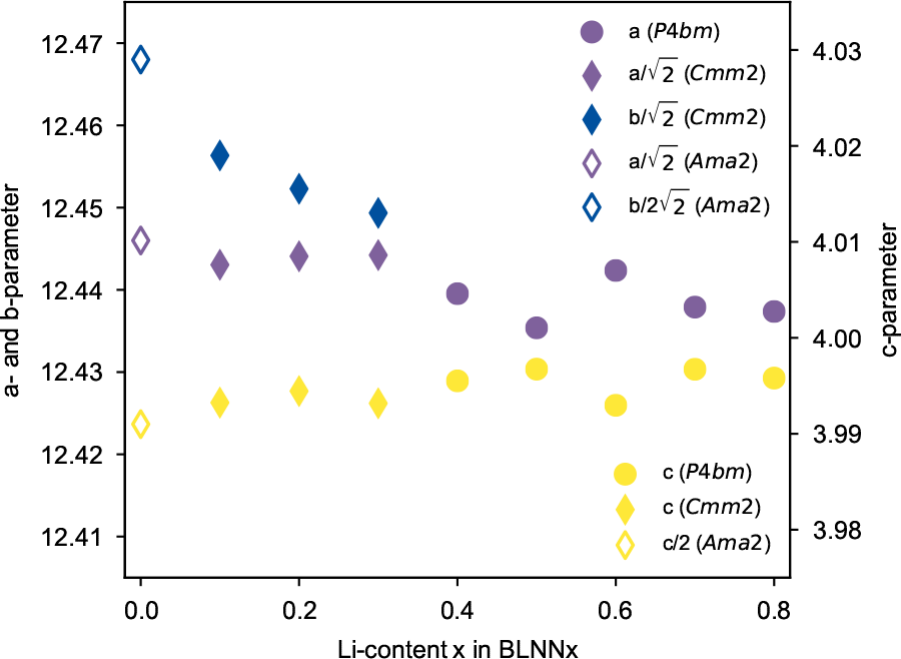
Lattice parameters as a function of the Li content, *x*, in the BLNN*x* compositions. Uncertainties associated with the plotted values are well within the size of the marker used.

The lattice parameter evolution of the BLNN solid solutions as a function of composition is presented in Figure 3. The values presented for BNN (*x* = 0) are from a previous work.^23^ With increasing Li content, *x*, from 0.10 to 0.30, the orthorhombic *b*-parameter decreases, while the *a*- and *c-*parameters show little change. From *x* of 0.30 to 0.50, the *a*- and *b*-parameters combine with the tetragonal *a*-parameter, which extends the trend of decline observed in the orthorhombic *b*-parameter. In the same range, the *c*-parameter slightly increases. At *x* = 0.60, the *a*-parameter increases and the *c*-parameter decreases before similar values as for *x* = 0.50 are obtained for *x* of 0.70 and 0.80.

The cation site occupation from Rietveld refinements of an ordered cation configuration and refinements that include adjustment of Tot_A2_ is demonstrated for BLNN50 (*x* = 0.50) in SI Figure S4. For the refinements where Tot_A2_ was set to 1, the ordered configuration yielded a poorer quality of fit than that for the refinements with allowed cation site disorder. The adjustment of Tot_A2_ was done manually in steps, decreasing the value from 1. Tot_A2_ needed to be manually adjusted as it would be 100% correlated with the Na occupancy on the A1-site (**Na**_**A1**_ in Table 1) if both parameters were to be freely refined at the same time. With varying Tot_A2_, the quality of the fit is the best (low GOF values), as well as constant, in the range Tot_A2_ = 1.0–0.93. A slight worsening of the fit is displayed when Tot_A2_ is set to 0.9, before a steady increase of the GOF value is seen on further lowering it (SI Figure S4a). When Tot_A2_ is decreased between 1 0 and 0.93 (SI Figure S4b,c), there is, virtually, only a decrease in the occupation of Na on the A2-site and a corresponding increase in the Na occupation on the A1-site. However, when Tot_A2_ is lowered beyond 0.93, the occupation of Na on the A2-site is already 0, and the Ba occupation on the A1-site increases to accommodate the restrictions of the model. The occupation of Li is only on C-sites, independent of Tot_A2_ (SI Figure S4d). The same effect was observed for all of the compositions. Tot_A2_ was therefore chosen to be constrained to 1 for all refinements for all of the compositions.

**Figure 4:**
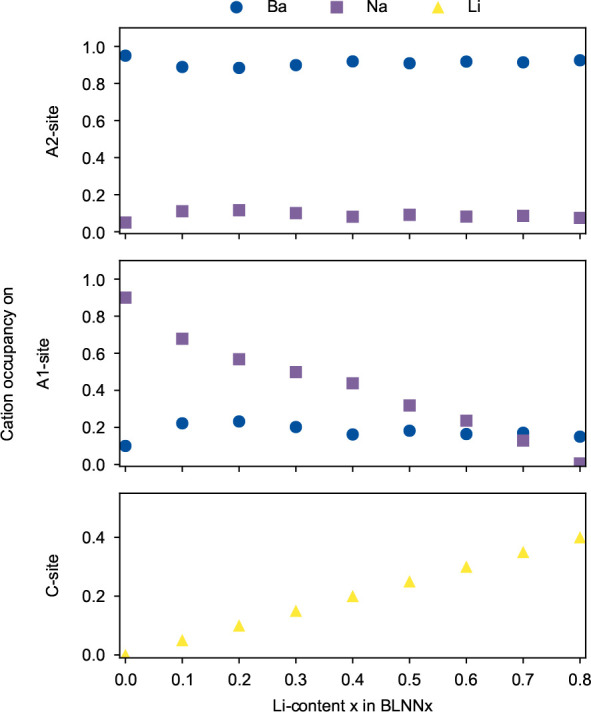
Cation site occupation on the (a) A2-, (b) A1-, and (c) C-sites as a function of the Li content *x* in BLNN solid solutions.

The cation site occupation resulting from the Rietveld refinements carried out with Tot_A2_ = 1, across the solid solution BLNN*x* (*x* = 0.10, 0.20, 0.30, 0.40, 0.50, 0.60, 0.70, and 0.80), are summarized in Figure 3 as well as in SI Tables S1–S8. The values presented for BNN (*x* = 0) are from a previous work.^23^ All Li in the solid solutions occupy the C-site, while both Ba and Na are distributed on both the A1- and A2-sites. Apart from slight changes between the Li content for *x* equal to 0 and 0.10 and between 0.30 and 0.40, which are compositional intervals where a change of space group symmetry used in refinement also occurs, the distribution of Ba and Na on the A2-sites (see Figure 4a) is constant across the whole compositional range. Thus, as the amount of Ba is constant for all compositions, the Ba occupation on the A1-site (Figure 4b) is also stable. When Na is gradually substituted by Li across the compositional range, a linear decrease of Na on the A1-site and a linear increase of Li on the C-sites with increasing Li content are seen. The values obtained for occupation of Li-ions exhibit relatively large error values compared to occupational values for Ba- and Na-ions (SI Tables S1–S8). However, as Li is a light element with a lower scattering contrast compared to the heavier Na- and Ba-ions, larger error values are expected. We also note that the relative error with respect to the Li content decreases with increasing Li content.

### DFT Calculations

**Figure 5:**
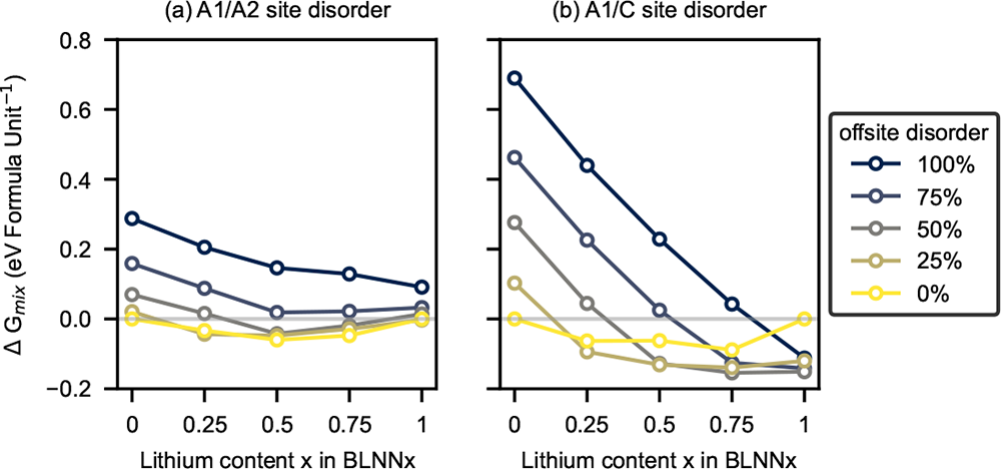
Gibbs free energies of mixing (Δ*G*_mix_) at 1000 K for solid solutions of BLNN including offsite disorder for (a) A1/A2-sites and (b) A1/C-sites, e.g., 25% A1/A2-site disorder in BNN (*x* = 0) corresponds to 75% Na on its nominal A1-site, while 50% A1/C-site disorder in BLN (*x* = 1) corresponds to 50% Li on the unoccupied C-site and 50% Li on the A1-site.

The Gibbs free energies of mixing (Δ*G*_mix_) for the solid solutions as a function of cation site disorder are given in Figure 5a,b for A1/A2- and A1/C-site disorder, respectively. The relative energies are given proportional to the endpoints (BNN and BLN) such that
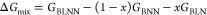
1

In terms of A1/A2-site disorder (Figure 5a) for *x* = 0 (BNN), offsite disorder is disfavored with Δ*G*_mix_ being the lowest in energy for 0% and rising to ∼0.3 eV/formula unit at 100% offsite disorder (corresponding to all Na existing on the A2-site and not on the nominal A1-site). This is in keeping with experimental observations and previous DFT calculations on BNN.^23^ Equally, for BNN, any A1/C-site disorder (Figure 5b) is heavily disfavored with 100% disorder between these two sites reaching ∼0.68 eV/formula unit.

**Figure 6:**
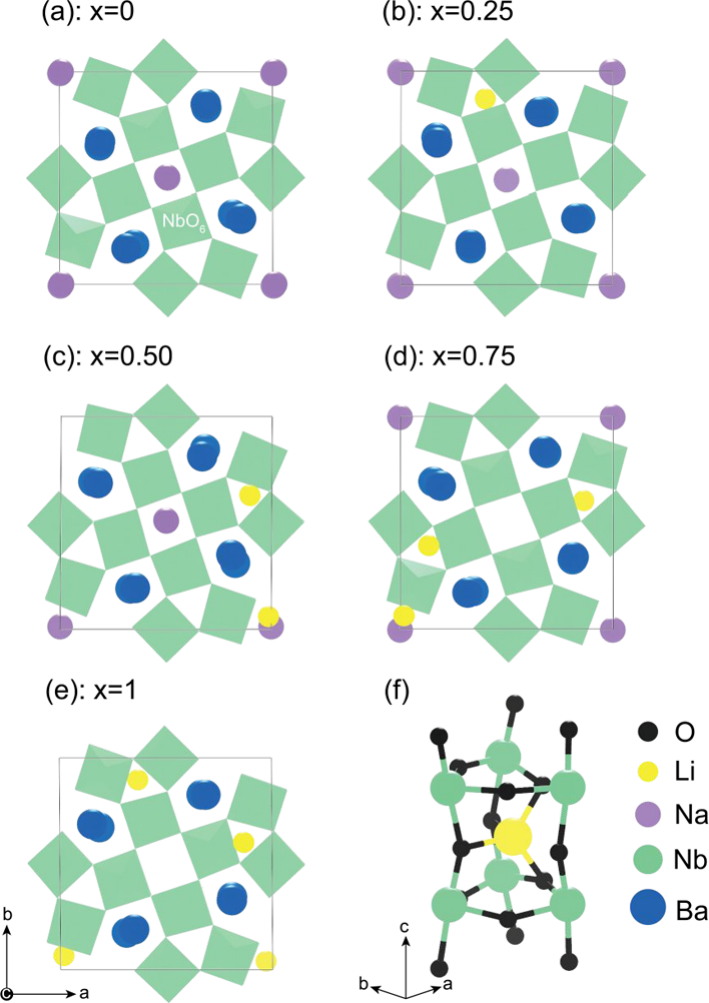
Representative lowest energy structures (relative to the ordered endpoints) for different *x* values of BLNN compositions: (a) = 0, (b) = 0.25, (c) = 0.5, (d) = 0.75, and (e) = 1. (f) Display of the preferential tetrahedral coordination of Li in the C-site.

For *x* = 1 (BLN), a different scenario is seen, with A1/C-site disorder being favored and A1/A2-site disorder being disfavored. For A1/C-site disorder in BLN, 0% offsite disorder is the highest in energy, while 50% site disorder is the most favorable. This result is understood due to the size difference between Na- and Li-ions, with the smaller Li preferring the smaller C-site in the TTB structure. Figure 6e gives the relaxed supercell as viewed along the c-direction, showing that 50% of the Li-ions occupy the C-sites and 50% occupy the A1-sites. When Li occupies these sites, it preferentially shifts off-center to form the LiO_4_ tetrahedra (Figure 6f), consistent with Li coordination in oxides in general.^43,44^

Solid solutions of BNN and BLN are expected to form for the entire compositional range, i.e., for *x* between 0 and 1; however, phase mixing is preferable at lower lithium contents *x* < 0.5 than at high Li contents, indicated by the flat energy landscape. In general, Li will always sit on the C-site, in a distorted off-center LiO_4_ configuration, and Na will always be on the A1-site. Increasing the Li content from BNN (*x* = 0) also makes 25 and 50% A1/A2-site disorder more favorable; however, with the exception of *x* = 0.25, 0% offsite disorder still gives the lowest energy configuration for BLNN due to Li’s preference for the C-site. It is still likely, however, that A1/A2-site disorder may be present in samples due to the negative Δ*G*_mix_, especially at low Li contents in BLNN. For *x* = 0.25 and 0.5, 25% offsite A1/C disorder is expected, while at *x* = 0.75, 50% A1/C-site disorder is the most preferential configuration. At a higher Li content (*x* = 0.75), it can be seen in Figure 6d that A1-site vacancies are present within the structure.

**Figure 7:**
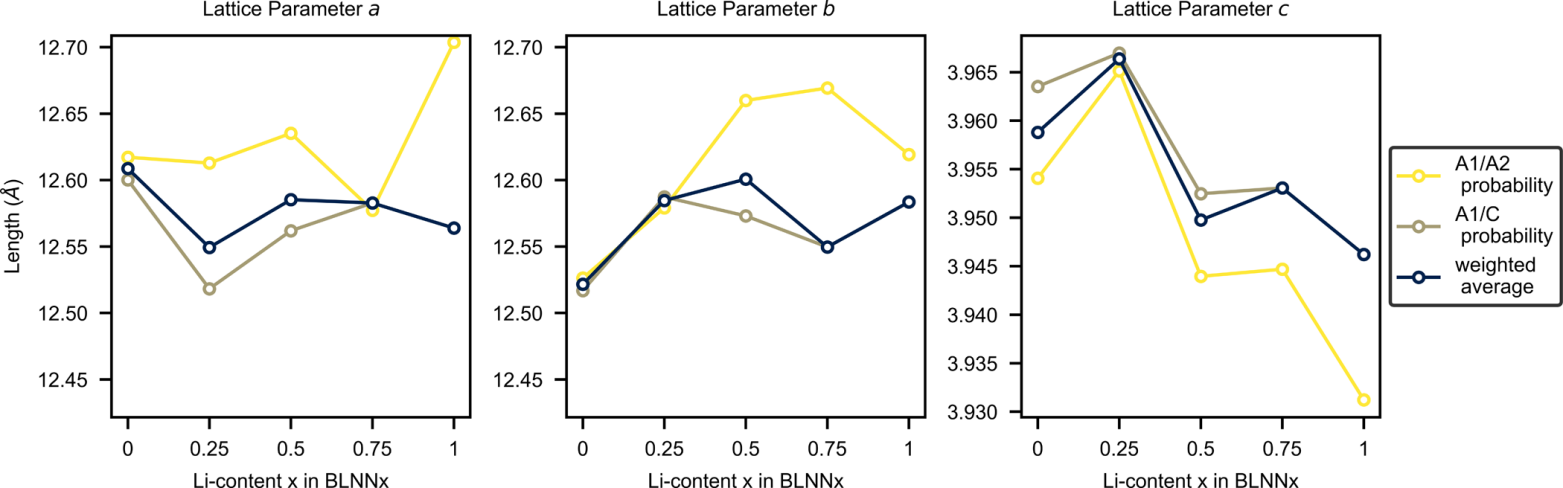
Probabilistic lattice parameters for BLNN solid solutions. Yellow lines correspond to structures where only A1/A2 cation disorder is acknowledged, whereas the light-brown line corresponds to those with only A1/C cation disorder. The blue line shows the weighted average of both where the weightings are chosen from the structures with an energy <0.08 eV (∼1000 K) of the lowest energy configuration.

Figure 7 shows the probabilistic lattice parameters for the BLNN solid solutions as a function of the A1/A2 and A1/C disorders and a weighted average of the two. Since negative Δ*G*_mix_ values exist for the A1/A2-sites, the weighted average of the two is calculated by taking into account the energies of both, where the weightings are chosen from the structures with an energy <0.08 eV (∼1000 K) of the lowest energy configurations. In general, the *c*-parameter changes the most upon incorporation of Li, reducing the magnitude significantly, while *a*- and *b*-parameters decrease and increase slightly with increasing Li content. At *x* = 0.25, the *c*-parameter reaches a maximum due to occupation on the C-site as well as the majority of the A1-site, which then tapers off with increasing *x* due to increased A1/C-offsite disorder. This is likely to significantly affect the *T*_C_ of the material, which relies on an out-of-plane shifting of the Nb–O octahedra.^24^

### Dielectric Properties

**Figure 8:**
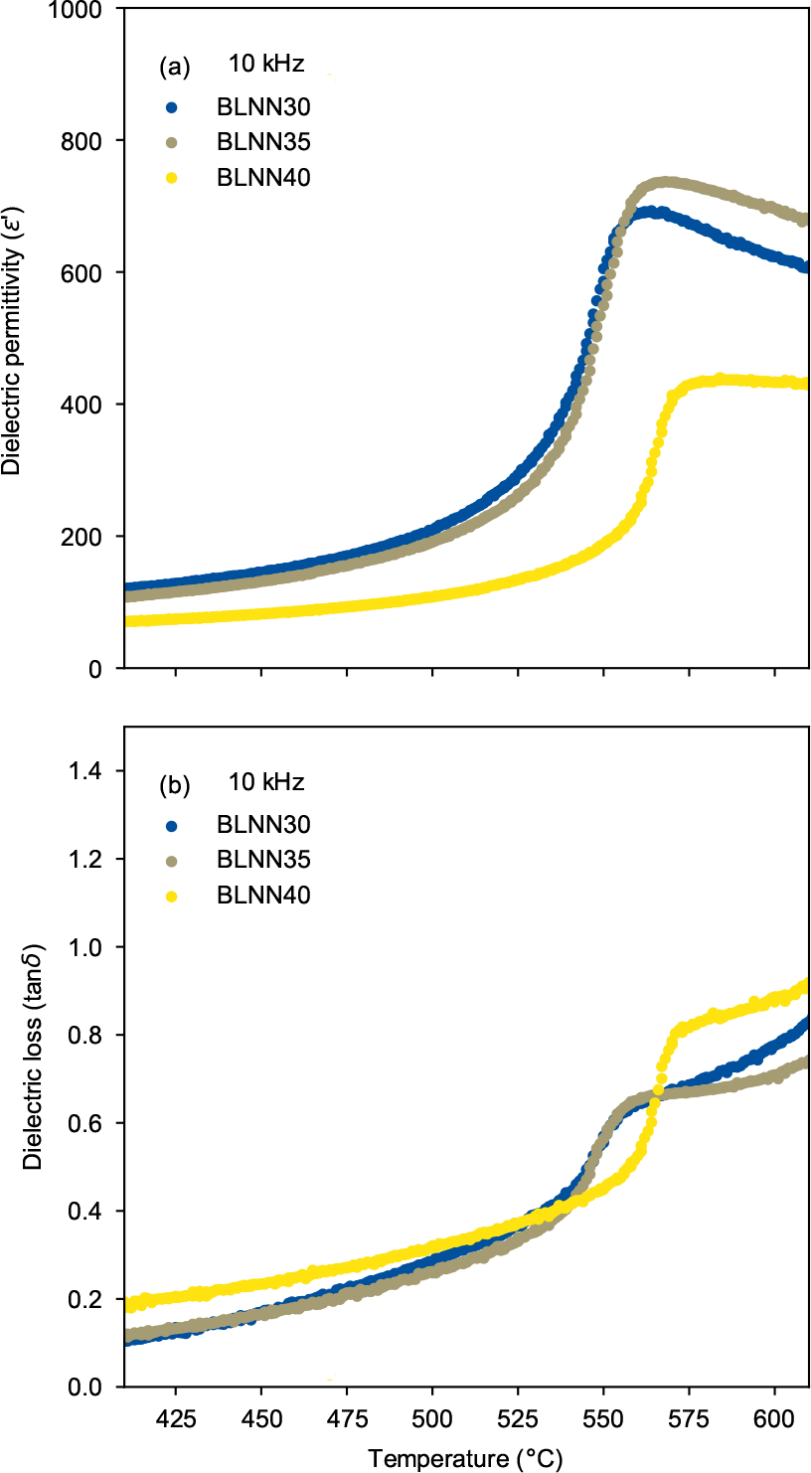
Temperature-dependent dielectric (a) permittivity and (b) loss for BLNN30, BLNN35, and BLNN40 at 10 kHz.

The temperature-dependent dielectric permittivity (ε′) and loss (tan δ) for BLNN30 (*x* = 0.30), BLNN35 (*x* = 0.35), and BLNN40 (*x* = 0.40) at 10 kHz are displayed in Figure 8. Dielectric permittivity values at 100 Hz, 1 kHz, and 10 kHz for each of the three solid solutions are shown in SI Figure S5. The pellets that were measured had relative densities of 87 (BLNN30), 89 (BLNN35), and 90% (BLNN40). Peak permittivity, corresponding to *T*_C_, above which the structure is expected to transition to the nonpolar TTB aristotype structure *P*4*/mbm*, is observed at ∼560 °C for BLNN30 and BLNN35 and at ∼570 °C for BLNN40. The observed peak permittivity values are about 7, 7.5, and 6.3 times larger than the room-temperature value for BLNN30, BLNN35, and BLNN40, respectively.

## Discussion

### Cation Site Occupation

The cation site occupation, which has been modeled by Rietveld refinement (Figure 4), based on a set of assumptions, shows that Li-ions prefer and occupy virtually only the C-sites of the TTB structure, that Na-ions occupy mainly the A1-sites, and that Ba-ions mainly occupy the A2-sites, which is in line with expectations from the literature.^45^ For BNN, where all A-sites are filled, some cross-occupation between Na and Ba is observed.^22,46,47^ The Ba configuration observed in the BLNN solid solutions is more or less constant independent of the Na-to-Li ratio and more interesting with an increase of the A-site vacancies because of Li sitting on C-sites. The observed stable configuration of Ba on both A1- and A2-sites, independent of the A-site vacancy concentration, is in contrast to what has recently been reported for the unfilled TTB (Sr,Ba)_5_Nb_10_O_30_ (SBN), where Ba is reported to only occupy the A2-sites.^41^ The authors suggested that the preference of Ba on A2-sites is fulfilled in SBN because of the A-site vacancies present, in contrast to BNN, where cross-occupation of Na and Ba occurs. The disorder in BNN was rationalized by restricted mobility due to the lack of site vacancies, locking in a disordered state in BNN. However, as Ba is seen to occupy both A1- and A2-sites, despite site vacancies being available to create mobility to settle in a different configuration, it appears that the configuration with approximately 90% of all Ba occupying the A2-site and 10% occupying the A1-site (occupancy of Ba on A1 is 20%) is preferred in the BLNN solid solutions.

In general, the results on cation site occupation from DFT calculations correspond well with the experimental results. In pure BNN, zero A1/C-site disorder is favorable, as expected (Figure 5b). This agrees with the constraint that only Li is allowed to move into the C-sites applied in the Rietveld refinements. For *x* of 0.25 and 0.50, the most energetically favored level of A1/C-site disorder corresponds to the Li content in the structure (i.e., 25 and 50%), indicating that all Li occupy the C-sites, while all Na occupy the A1-sites, in perfect agreement with the experimental results. For *x* = 0.75 and *x* = 1 (BLN), it is still the occupation of 50% site disorder that is the most energetically favored. When one assumes that only Li occupies the C-sites, this result indicates that there is a mix of Li and Na on the A1-sites. This is in contrast with the experimental results for *x* of 0.70 and 0.80; however, as minor traces of secondary phases are present in these compositions, the stoichiometry is off, and aberrations are therefore expected. Additionally, it should be noted that the DFT calculations do not take into account the nonstoichiometry or potential effects from point defects and there may be limited interaction between periodic images due to finite supercell effects.

Zero A1/A2-site disorder is the most energetically favorable state for *x* = 0 (BNN) (Figure 5a). From the experiment, cross-occupation giving 12% Ba on the A1-site is expected^23^ (Figure 4); however, as the compositional resolution of the DFT calculations is in steps of 0.25, we can only conclude that no disorder is more favorable than Ba occupation on the A1-site of 25% or more. Also, for *x* = 1 (BLN), it is the most energetically favorable with no disorder. This indicated that it is energetically unfavorable for Li to occupy the A2-site, which agrees with our choice of constraint in the Rietveld refinements, only allowing Ba and Na to occupy the A2-site. For the solid solution composition of *x* = 0.25, the occupation of 25% Ba on the A1-site is the most energetically favored. This corresponds well to the higher Ba occupation on the A1-site observed experimentally for the BLNN solid solutions. For *x* of 0.5 and 0.75, it is the 0% Ba on A1 that is the most favored; however, the energy landscape between 0, 25, and 50% is very flat, indicating that cross-occupation, as seen experimentally, in Figure S4a, to be preferred compared to the ordered configuration is not unexpected.

Ultimately, we note that absolute determination of the occupancy of four species (Ba, Na, Li, and vacancies) on three distinct sites is impossible with the data we have available. It is because we have assumed certain constraints of which sites certain cations were allowed to occupy as well as having assumed a certain degree of occupation on the A2-site, which allowed us to retrieve the current results.

### Phase Transition and Lattice Parameters

BNN and BLN are reported to have orthorhombic and tetragonal symmetries, respectively. Thus, an increase of the symmetry is expected across the solid solution BLNN*x* as *x*, the Li content, is increased by a one-to-one substitution by Na. From XRD and Pawley refinement, the transition is suggested to occur at a composition between 0.30 and 0.35 (Figure 1 and SI Figure S1).

We have previously hypothesized that a shift from orthorhombic to tetragonal symmetry in filled Ba-based TTBs is governed by the average size of the cations occupying the A1-site and that a mean size of cation occupying the A1-site smaller than some minimum value induces orthorhombic symmetry.^23^ In the BLNN solid solutions, however, we see a shift from orthorhombic to tetragonal symmetry as smaller cations are introduced. Given a constant Ba configuration, as Li-ions occupy the C-sites, the average size of the cations occupying the A1-site actually increases, in line with the previous suggestion. This is true when disregarding the effect of the increasing vacancy concentration, as the Ba/Na ratio on the A1-site increases. However, the effect of the increased vacancy concentration on the A-site in TTBs is not clear, and it is therefore likely that this relation is more complex in the BLNN solid solutions.

The tetragonal *P*4*bm* symmetry, reported for BLN, has been assigned to the BLNN*x* compositions with *x* ≥ 40. It is worth mentioning that there is no change in the allowed reflections between the centrosymmetric aristotype TTB structure *P*4*/mbm* and the noncentrosymmetric *P*4*bm*. However, clear maxima in the temperature-dependent permittivity, indicating a paraelectric–ferroelectric transition for the solid solutions with *x* of 0.30, 0.35, and 0.40 (Figure 8 and SI Figure S5), are observed. Additionally, no distinct contraction in the *c*-parameter, which often accompanies a ferroelectric–paraelectric transition in TTBs, is observed across the compositional range. Thus, the *P*4*bm* symmetry has been assigned over *P*4*/mbm*.

Two recent studies by Whittle et al.,^48^ based on literature and group theory, and by Grendal et al.,^46^ based on HR-XRD, have concluded that BNN is best modeled by the *Ama*2 (standard setting of *Bbm*2) space group at room temperature. In the diffraction data, the determination of the *Ama*2 space group is done based on the observation of weak satellite reflections (especially visible around 2.8 Å^–1^), which is not allowed in *Cmm*2. The HR-XRD used in the analysis of Grendal et al. is collected at the same beamline (ID 22, ESRF) as our HR-XRD. However, it is the orthorhombic *Cmm*2 symmetry that appears to give the best model of BLNN10–35 (Figure 2 and SI Figure S1) as we do not observe the same satellite reflections. This indicates that upon substitution of 10% Na for Li, the doubling of the *c*-parameter and tilt patterns associated with the *Ama*2 symmetry is no longer stabilized,^48−50^ or at the very least, the effect is considerably weaker.

In the literature, single crystals of BLN are reported to be grown with a slight excess of Ba in order to stabilize the TTB structure.^20^ It is thus expected that a stability limit exists when moving toward pure BLN and that a secondary phase is introduced. The first trace of a secondary phase is observed for *x* = 0.70 but becomes more prominent for higher Li contents (SI Figure S2a). While no explicit thermodynamic or dynamic stability calculations have been performed using DFT, the flat energy landscape between BNN and BLN from *x* = 0.25 to *x* = 1 (Figure 5b) (a lower free energy is observed for *x* = 0.75 than for *x* = 1 by 0.003 eV per formula unit (SI Table S12)) indicates that BLN is not necessarily as stable as the solid solutions, within the constraints of our computational model. As only the aristotype *P*4*/mbm* TTB structure was used as the basis for the DFT, and due to the finite size of the supercell, we cannot rule out that additional structures/symmetries may be more favorable and thus phase separation was favored.

The evolution of the lattice parameter as a function of the Li content (Figure 3) reflects both the phase transitions and the stability limit that is exhibited in the BLNN*x* solid solution system. First, a decrease in the *a-* and *b*-parameters is seen from *x* of 0 (BNN, values from^23^) to 0.10, where we expect a transition from *Ama*2 to *Cmm*2 symmetry. Then the merging of the *a*- and *b*-parameters occurs upon the transition from orthorhombic *Cmm*2 to tetragonal *P*4*bm*. The *c*-parameter shows little variation across the two phase transitions, which could indicate that no ferroelectric–paraelectric transition occurs. Additionally, a kink is seen in both the *a*- and *b*-parameters as a function of Li content, for a Li content ranging from 0.50 to 0.70. This is interpreted as a sign of a frustrated lattice as the structure approaches a stability limit. As the lattice parameter evolution obtained from DFT calculations (Figure 7) has a different compositional resolution than from experimental data, the comparison is not straightforward. However, the splitting in *a*- and *b*-parameters, indicating a transition to an orthorhombic space group for low Li contents, seems to be captured by the weighted average of the probabilistic lattice parameters achieved from structures where A1/A2-site and A1/C-site disorder was allowed separately with DFT.

The stability limit of the TTB structure can possibly be understood via the cation occupation and especially with respect to the vacancy concentration on the A1-site, which increases with increasing Li content as all Li cations occupy the C-sites. As demonstrated for BLNN50 (*x* = 0.50), the quality of fit is constant in the range Tot_A2_ of 1.0–0.93 (SI Figure S4). This means that we cannot conclude a distinct cation configuration on the A-sites. However, the maximum possible occupation on the A1-site, when considering cases that do not give a considerable worsening in the Rietveld refinement, is achieved at a Tot_A2_ of 0.9. This gives a maximum total occupation of cations on the A1-site (Tot_A1_) of 55% when *x* = 0.60. To achieve phase compositions with *x* of 0.70 and 0.80, vacancy concentrations of at least 55 and 65% would have to be tolerated, but this is clearly not the case as secondary phases are introduced. For comparison, the vacancy concentration seen in SBN with a Sr fraction of 0.25 is ∼40%, which is in between the minimum values when *x* is 0.50 and 0.60.^41^

### The Curie Temperature

The *T*_C_ values for filled Ba-based Ba_4_M_2_Nb_10–2*x*_Ti_2*x*_O_30_ TTBs are reported to increase with decreasing size of the M-cation.^23,51^ In this work, the M-cation corresponds to Na and Li, and thus, the size of M decreases with increasing Li content. The *T*_C_ for BLNN*x* with *x* of 0.30, 0.35, and 0.40 is within the range reported for BNN at 550–580 °C.^19,47^ Relative size differences between A-cations and disorder are known factors with respect to the *T*_C_. A slight increase in the *T*_C_ is seen with increasing Li content, but as the Li-ions have only a minor influence on the configuration of A-cations and the level of disorder in the system, it is not surprising that a large change in *T*_C_ is not observed. Whether BLNN exhibits enhanced electrical or electromechanical properties across this tetragonal-to-orthorhombic phase transition requires further examination. However, higher density samples are required to perform robust electrical characterization, a factor that prohibited the inclusion of such a study in this work, and thus, density optimization is first required.

## Conclusions

Phase-pure solid solutions of BNN and BLN were achieved up to 60% BLN. Structural assessments revealed that with 10–30% Li substituted for Na, the *Cmm*2 structure gives the best model at room temperature, in contrast to pure BNN, which is best described by *Ama*2 symmetry. An orthorhombic-to-tetragonal (*P*4*bm*) transition was identified to occur in the range of 30–35% Li. Cation site occupation on the A1-, A2-, and C-sites modeled with Rietveld refinement revealed that the Ba configuration is constant across the compositional range, with approximately 90% of all Ba occupying the A2-site and 10% of all Ba occupying the A1-site. The Na occupancy on A2- and A1-sites depends on the total concentration of Na in each composition, while Li has a preference to occupy the C-sites. DFT calculations are generally in agreement with the site occupancies obtained from the experimental data. The *T*_C_ across the orthorhombic-to-tetragonal phase transition was identified at maxima in temperature-dependent permittivity and was observed to be comparable to what has been reported for BNN.

## Supporting Information Available

The following files are available free of charge. X-ray diffraction patterns of BLNN*x* (*x* = 0.10, 0.20, 0.30, 0.40, and 1.0) with Pawley refined patterns overlaid; X-ray diffraction patterns of BLNN*x* (*x* = 0.70, 0.80, 0.85, 0.90, and 1.0); illustrations of TTB and “TTB-type” structures; cation occupation in BLNN50 (*x* = 0.50); temperature-dependent permittivity for BLNN*x* (*x* = 0.30, 0.35, and 0.40); and details of Rietveld refinements and DFT calculations (PDF)
