# Supplementary material for: Crystal Structure, Cation Occupation, and Phase Transitions in Ba4(LixNa1–x)2Nb10O30 Tetragonal Tungsten Bronzes
Source: Inorg Chem. 2025 Jan 3;64(2):1031–40. doi: 10.1021/acs.inorgchem.4c04461 (PMC11752527; doi:10.1021/acs.inorgchem.4c04461)
Supplement: Supplementary file 1 — ic4c04461_si_001.pdf [file ic4c04461_si_001.pdf]

Supporting information: Crystal structure, cation occupation and phase transitions in $Ba_4(Li_xNa_{1-x})_2Nb_{10}O_{30}$ tetragonal tungsten bronzes

Nora Statle Løndal, Benjamin A. D. Williamson, Ola G. Grendal, Julian Walker, Mari-Ann Einarsrud and Tor Grande*

Department of Material Science and Engineering, NTNU Norwegian University of Science and Technology,

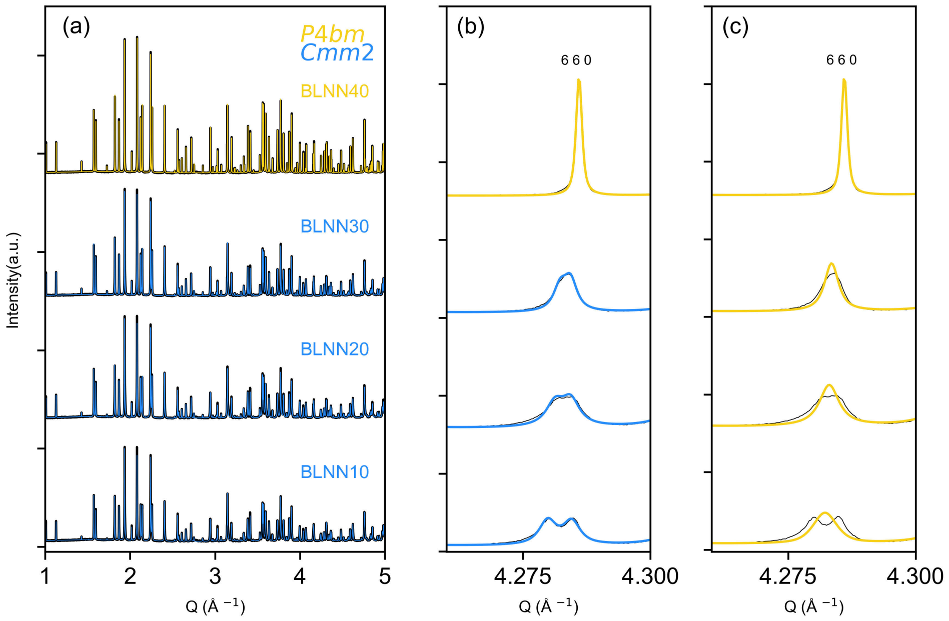

Figure S1: Pawley refinement of HR-XRD pattern of BLNN10, BLNN20, BLNN30 and BLNN40 (x = 0.10, 0.20, 0.30 and 0.40) with the space groups *P4bm and Cmm*2 at (a) 1-5 Å^{-1} as well as at (b, c) 4.26-4.30 Å^{-1}. BLNN10, BLNN20 and BLNN3 show good fit with the *Cmm*2 symmetry model, which capture the observed splitting of the 660-reflection, while the tetragonal *P4bm* symmetry is sufficient to model BLNN40.

* Author to whom correspondence should be addressed: tor.grande@ntnu.no

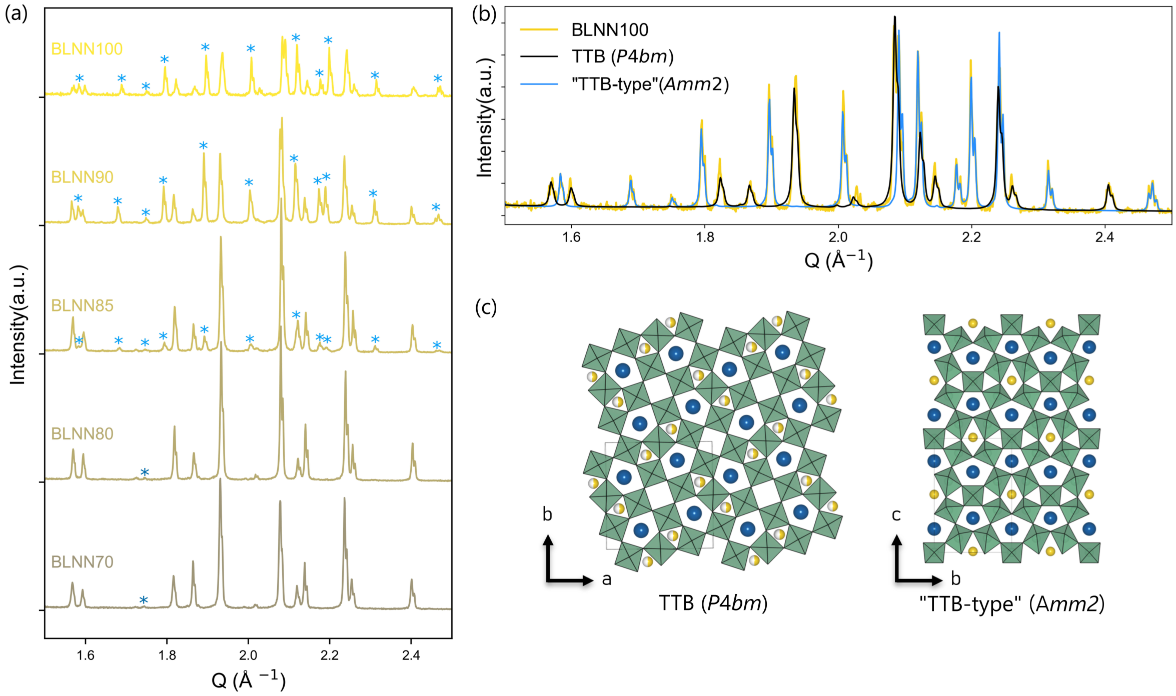

Figure S2: A secondary phase is observed in BLNN solid solutions with high Li-content. (a) F-XRD patterns for BLNN70-BLNN100 (BLN), where reflections not described by the TTB structure is marked with asterisks. (b) Pawley refinement of BLNN100 F-XRD patterns (yellow line), where the modelled TTB structure (*P*4*bm*) and secondary phase "TTB-type" structure (*Amm*2) is overlaid the diffraction pattern with black and blue lines, respectively. (c) Illustrations of the TTB (*P*4*bm*) and "TTB-type" (*Amm*2) structures.

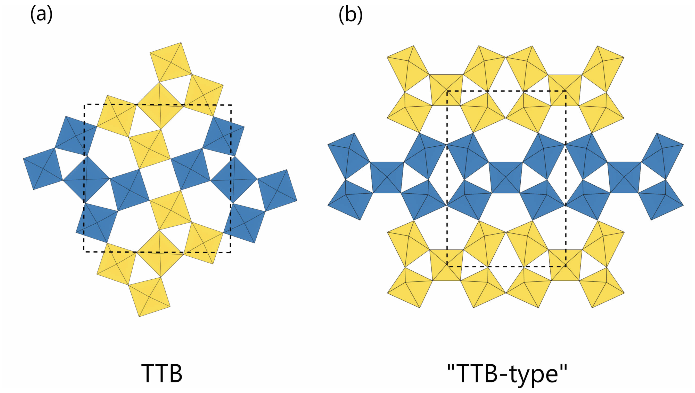

Figure S3: The building block of the (a) TTB structure and the (b) "TTB-type" structure is the same block of five corner sharing NbO_6-octahedra, but the building blocks are organised differently in the two. Both structures have trigonal (present in the building block itself), quadrilateral and pentagonal sites. In the TTB structure the quadrilateral sites are square sites built from octahedra of four distinct building blocks, while in the "TTB-type" structure these are deltoidal, built from two distinct building

blocks. Yellow and blue colour is used here for better visibility, but all blocks within each structure is equal.

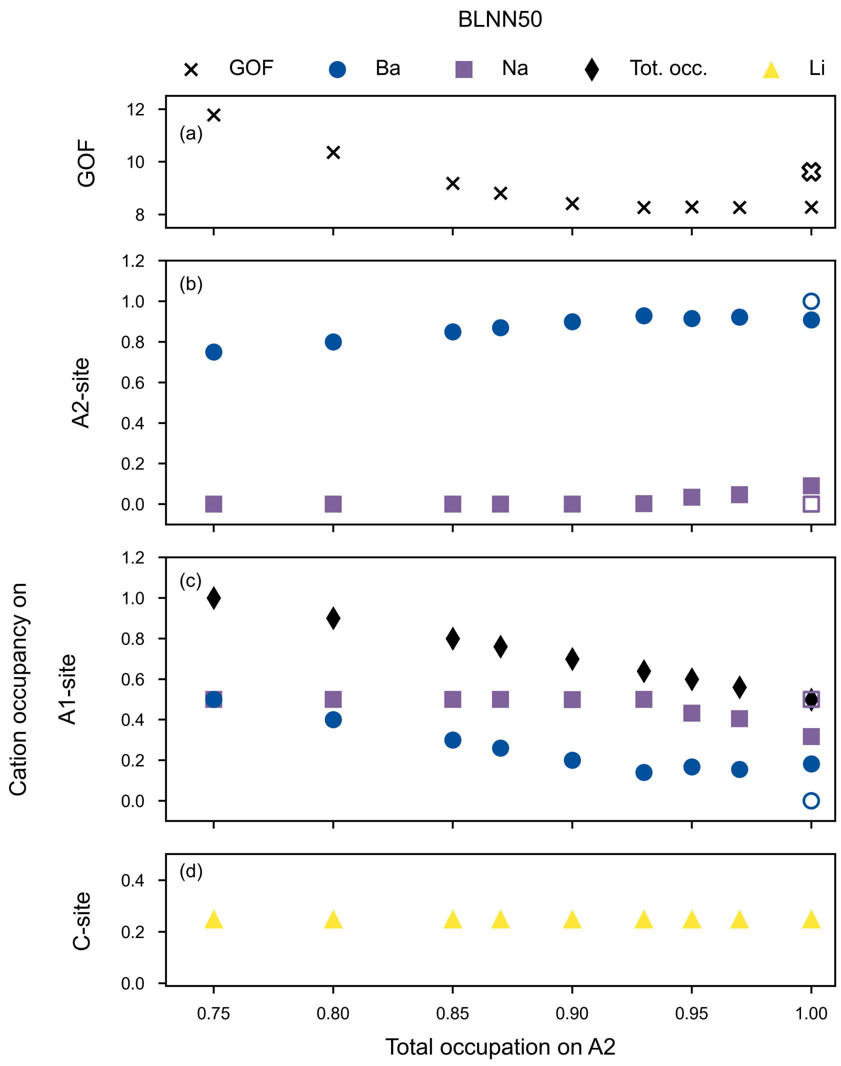

Figure S4: The quality of fit (a) “Goodness of fit” (GOF)- values and cation site occupation on the (b) A2-, (c) A1- and (d) C-site in BLNN50 as function of the total occupation on the A2-site (T_{occA2}). Unfilled markers are used for refinement of an ordered configuration where Ba occupies the A2-sites, Na occupies the A-sites and Li occupies C-sites.

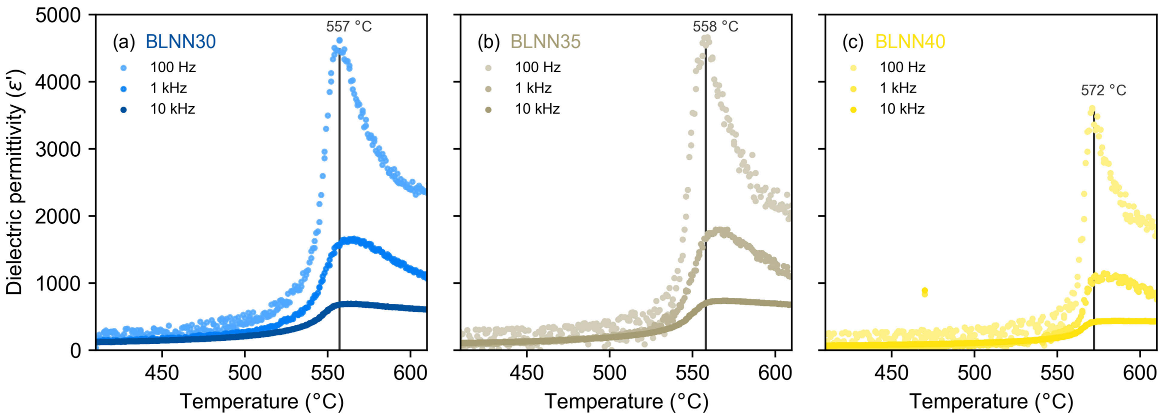

Figure S5: Temperature dependent dielectric permittivity for (a) BLNN30 (x=0.30), (b) BLNN35 (x=0.35) and (c) BLNN40 (x = 0.40) at 100 Hz, 1 kHz and 10 kHz.

Table S1. Refined *Cmm*2 crystal structure of $Ba_4(Na_{0.9}Li_{0.1})_2Nb_{10}O_{30}$. The number of independent parameters that were refined was 36. Unit cell parameters: a = 17.596934(61) Å, b = 17.616061(63) and c = 3.993270(12) Å. GOF: 6.59, Rwp: 12.74 Refined parameters are in bold.

Site	Mp	Atom	X	Y	Z	Occupancy	B_{eq}
A1	4	Na	0.2500	0.7500	**0.549(1)**	**0.685(3)**	1.6
		Ba				**0.215(3)**	1.6
		Li				**0.000(54)**	1.6
A2_1	4	Ba	0.5000	**0.1722(1)**	0.5000	**0.893(1)**	0.44
		Na				**0.107(1)**	0.44
A2_2	4	Ba	**0.8273(1)**	0.0000	0.5000	**0.893(1)**	0.44
		Na				**0.107(1)**	0.44
C1	4	Li	0.0000	0.1200	0.5000	**0.05(3)**	1
C2	4	Li	0.8800	0.5000	0.5000	**0.05(3)**	1
Nb1_1	2	Nb	0.5000	0.0000	**0.012(4)**	1	0.42
Nb2_1	2	Nb	0.0000	0.0000	**0.013(4)**	1	0.42
Nb2_1	8	Nb	**0.18206(8)**	**0.39177(7)**	**0.011(2)**	1	0.42
Nb2_1	8	Nb	**0.60824(8)**	**0.68215(8)**	**0.011(2)**	1	0.42
O1_1	4	O	0.5000	0.2847	0.9550	1	1.17
O1_2	4	O	0.7170	0.0000	0.9664	1	1.17
O2_1	8	O	0.3584	0.2130	0.9328	1	1.17
O2_2	8	O	0.7865	0.8525	0.9459	1	1.17
O3_1	8	O	0.0736	0.4168	-0.0653	1	1.17
O3_2	8	O	0.5800	0.5738	-0.0494	1	1.17
O4_1	8	O	0.5000	0.0000	0.4241	1	1.17
O4_2	8	O	0.0000	0.0000	0.5330	1	1.17
O5_1	2	O	0.1929	0.3935	0.4574	1	1.17
O5_2	2	O	0.6166	0.6735	0.4636	1	1.17

Table S2. Refined *Cmm*2 crystal structure of $Ba_4(Na_{0.8}Li_{0.2})_2Nb_{10}O_{30}$. The number of independent parameters that were refined was 36. Unit cell parameters: a = 17.596304(74) Å, b = 17.609879(78) Å and c = 3.994251(14) Å. GOF: 8.13, Rwp: 14.6294121. Refined parameters are in bold.

Site	Mp	Atom	X	Y	Z	Occupancy	B_{eq}
A1	4	Na	0.2500	0.7500	**0.560(1)**	**0.567(3)**	1.6
		Ba				**0.233(3)**	1.6
		Li				**0.00(6)**	1.6
A2_1	4	Ba	0.5000	**0.1749(1)**	0.5000	**0.884(2)**	0.44
		Na				**0.116(2)**	0.44
A2_2	4	Ba	**0.8302(1)**	0.0000	0.5000	**0.884(2)**	0.44
		Na				**0.116(2)**	0.44
C1	4	Li	0.00000	0.12000	0.50000	**0.10(3)**	1
C2	4	Li	0.88000	0.50000	0.50000	**0.10(3)**	1
Nb1_1	2	Nb	0.50000	0.00000	**0.0140(4)**	1	0.42
Nb2_1	2	Nb	0.00000	0.00000	**0.016 (4)**	1	0.42
Nb2_1	8	Nb	**0.1821(1)**	**0.3919(1)**	**0.014(2)**	1	0.42
Nb2_1	8	Nb	**0.6083(1)**	**0.6821(1)**	**0.015(2)**	1	0.42
O1_1	4	O	0.5000	0.2847	0.9550	1	1.17
O1_2	4	O	0.7170	0.0000	0.9664	1	1.17
O2_1	8	O	0.3584	0.2130	0.9328	1	1.17
O2_2	8	O	0.7865	0.8525	0.9459	1	1.17
O3_1	8	O	0.0736	0.4168	-0.0653	1	1.17
O3_2	8	O	0.5800	0.5738	-0.0494	1	1.17
O4_1	8	O	0.5000	0.0000	0.4241	1	1.17
O4_2	8	O	0.0000	0.0000	0.5330	1	1.17
O5_1	2	O	0.1929	0.3935	0.4574	1	1.17
O5_2	2	O	0.6166	0.6735	0.4636	1	1.17

Table S3. Refined *Cmm*2 crystal structure of $Ba_4(Na_{0.7}Li_{0.3})_2Nb_{10}O_{30}$. The number of independent parameters that were refined was 36. Unit cell parameters: a = 17.601942(156) Å, b = 17.601813(155) Å and c = 3.992942(12) Å. GOF: 7.25, Rwp: 14.5395062. Refined parameters are in bold.

Site	Mp	Atom	X	Y	Z	Occupancy	B_{eq}
A1	4	Na	0.2500	0.7500	**0.443(2)**	**0.510(3)**	1.6
		Ba				**0.190(3)**	1.6
		Li				**0.000(59)**	1.6
A2_1	4	Ba	0.5000	**0.16754 (9)**	0.5000	**0.905(2)**	0.44
		Na				**0.095(2)**	0.44
A2_2	4	Ba	**0.82264(9)**	0.0000	0.5000	**0.905(2)**	0.44
		Na				**0.095(2)**	0.44
C1	4	Li	0.0000	0.1200	0.5000	**0.150(30)**	1
C2	4	Li	0.8800	0.5000	0.5000	**0.150(30)**	1
Nb1_1	2	Nb	0.5000	0.0000	**-0.018 (4)**	1	0.42
Nb2_1	2	Nb	0.0000	0.0000	**-0.021 (3)**	1	0.42
Nb2_1	8	Nb	**0.1815 (1)**	**0.3920(1)**	**-0.020 (2)**	1	0.42
Nb2_1	8	Nb	**0.6086 (1)**	**0.6826(1)**	**-0.021 (2)**	1	0.42
O1_1	4	O	0.5000	0.2847	0.9550	1	1.17
O1_2	4	O	0.7170	0.0000	0.9664	1	1.17
O2_1	8	O	0.3584	0.2130	0.9328	1	1.17
O2_2	8	O	0.7865	0.8525	0.9459	1	1.17
O3_1	8	O	0.0736	0.4168	-0.0653	1	1.17
O3_2	8	O	0.5800	0.5738	-0.0494	1	1.17
O4_1	8	O	0.5000	0.0000	0.4241	1	1.17
O4_2	8	O	0.0000	0.0000	0.5330	1	1.17
O5_1	2	O	0.1929	0.3935	0.4574	1	1.17
O5_2	2	O	0.6166	0.6735	0.4636	1	1.17

Table S4. Refined *P4bm* crystal structure of $Ba_4(Na_{0.6}Li_{0.4})_2Nb_{10}O_{30}$. The number of independent parameters that were refined was 31. Unit cell parameters: a = 12.439505(9) Å and c = 3.995505(4) Å. GOF: 6.87, Rwp: 14.12. Refined parameters are in bold.

Site	Mp	Atom	X	Y	Z	Occupancy	B_{eq}
A1	2	Na	0.0000	0.0000	**0.4439(1)**	**0.409 (2)**	1.6
		Ba				**0.191(2)**	1.6
		Li				**0.00(6)**	1.6
A2	4	Ba	**0.67240(3)**	**0.17240(3)**	**0.4867(8)**	**0.905(1)**	0.44
		Na				**0.095(1)**	0.44
C	4	Li	0.1200	0.6200	0.5000	**0.20(3)**	1
Nb1	2	Nb	0.0000	0.5000	0.0059	1	0.42
Nb2	8	Nb	**0.57381(4)**	**0.70960(4)**	**0.0028(7)**	1	0.42
O1	8	O	0.78418	0.2842	-0.0365	1	1.17
O2	8	O	0.56984	0.3577	0.9286	1	1.17
O3	4	O	0.49234	0.8421	0.0556	1	1.17
O4	8	O	0.50000	0.0000	0.4311	1	1.17
O5	2	O	0.57357	0.7058	0.4451	1	1.17

Table S5. Refined *P4bm* crystal structure of $Ba_4(Na_{0.5}Li_{0.5})_2Nb_{10}O_{30}$. The number of independent parameters that were refined was 31. Unit cell parameters: a = 12.435944 (11) Å and c = 3.996927 (5) Å. GOF: 8.79, Rwp: 17.71. Refined parameters are in bold.

Site	Mp	Atom	X	Y	Z	Occupancy	B_{eq}
A1	2	Na	0.00000	0.00000	**0.434(1)**	**0.299 (3)**	1.6
		Ba				**0.201 (3)**	1.6
		Li				**0.00(7)**	1.6
A2	4	Ba	**0.67239(4)**	**0.17239(4)**	**0.485(1)**	**0.900(2)**	0.44
		Na				**0.100(2)**	0.44
C	4	Li	0.1200	0.6200	0.50000	**0.25(3)**	1
Nb1	2	Nb	0.0000	0.5000	**0.005(1)**	1	0.42
Nb2	8	Nb	**0.57380(5)**	**0.70959(5)**	**0.0019(9)**	1	0.42
O1	8	O	0.78418	0.2842	-0.0365	1	1.17
O2	8	O	0.56984	0.3577	0.9286	1	1.17
O3	4	O	0.49234	0.8421	0.0556	1	1.17
O4	8	O	0.50000	0.0000	0.4311	1	1.17
O5	2	O	0.57357	0.7058	0.4451	1	1.17

Table S6. Refined *P4bm* crystal structure of $Ba_4(Na_{0.4}Li_{0.6})_2Nb_{10}O_{30}$. The number of independent parameters that were refined was 31. Unit cell parameters: a = 12.442687 (10) Å and c = 3.993082 (4) Å. GOF: 7.80, Rwp: 15.85. Refined parameters are in bold.

Site	Mp	Atom	X	Y	Z	Occupancy	B_{eq}
A1	2	Na	0.00000	0.00000	**0.43(1)**	**0. 214(3)**	1.6
		Ba				**0.186 (3)**	1.6
		Li				**0.00(6)**	1.6
A2	4	Ba	**0.67231(3)**	**0.17231(3)**	**0.4857(9)**	**0.907(1)**	0.44
		Na				**0.093(1)**	0.44
C	4	Li	0.12000	0.62000	0.50000	**0.30(3)**	1
Nb1	2	Nb	0.00000	0.50000	**0.002(1)**	1	0.42
Nb2	8	Nb	**0.57383(4)**	**0.70921(4)**	**-0.0024(8)**	1	0.42
O1	8	O	0.78418	0.2842	-0.0365	1	1.17
O2	8	O	0.56984	0.3577	0.9286	1	1.17
O3	4	O	0.49234	0.8421	0.0556	1	1.17
O4	8	O	0.50000	0.0000	0.4311	1	1.17
O5	2	O	0.57357	0.7058	0.4451	1	1.17

Table S7. Refined *P4bm* crystal structure of $Ba_4(Na_{0.3}Li_{0.7})_2Nb_{10}O_{30}$. The number of independent parameters that were refined was 31. Unit cell parameters: a = 12.438055(10) Å and c = 3.996781 (4) Å. GOF: 7.30, Rwp: 14.94. Refined parameters are in bold.

Site	Mp	Atom	X	Y	Z	Occupancy	B_{eq}
A1	2	Na	0.00000	0.00000	**0.435(1)**	**0. 100 (3)**	1.6
		Ba				**0. 200 (3)**	1.6
		Li				**0.00(6)**	1.6
A2	4	Ba	**0.67235(4)**	**0.17235(4)**	**0.4815(9)**	**0.900(1)**	0.44
		Na				**0.100(1)**	0.44
C	4	Li	0.12000	0.62000	0.50000	**0.35(3)**	1
Nb1	2	Nb	0.00000	0.50000	**0.002(1)**	1	0.42
Nb2	8	Nb	**0.57377(4)**	**0.70934(4)**	**-0.0020(8)**	1	0.42
O1	8	O	0.78418	0.2842	-0.0365	1	1.17
O2	8	O	0.56984	0.3577	0.9286	1	1.17
O3	4	O	0.49234	0.8421	0.0556	1	1.17
O4	8	O	0.50000	0.0000	0.4311	1	1.17
O5	2	O	0.57357	0.7058	0.4451	1	1.17

Table S8. Refined *P4bm* crystal structure of $Ba_4(Na_{0.2}Li_{0.8})_2Nb_{10}O_{30}$. The number of independent parameters that were refined was 31. Unit cell parameters: a = 12.437297(10) Å and c = 3.995786(4) Å. GOF: 7.61, Rwp: 15.00. Refined parameters are in bold.

Site	Mp	Atom	X	Y	Z	Occupancy	B_{eq}
A1	2	Na	0.00000	0.00000	**0.433(1)**	**0.014(3)**	1.6
		Ba				**0.186(3)**	1.6
		Li				**0.00(6)**	1.6
A2	4	Ba	**0.67231(3)**	**0.17231(3)**	**0.4823(9)**	**0.907(1)**	0.44
		Na				**0.093(1)**	0.44
C	4	Li	0.1200	0.6200	0.5000	**0.40(3)**	1
Nb1	2	Nb	0.0000	0.5000	**0.002(1)**	1	0.42
Nb2	8	Nb	**0.57376(4)**	**0.70915(4)**	**-0.0013(8)**	1	0.42
O1	8	O	0.78418	0.2842	-0.0365	1	1.17
O2	8	O	0.56984	0.3577	0.9286	1	1.17
O3	4	O	0.49234	0.8421	0.0556	1	1.17
O4	8	O	0.50000	0.0000	0.4311	1	1.17
O5	2	O	0.57357	0.7058	0.4451	1	1.17

Thermodynamics of Cation Site Disorder

A1/A2/C cation site disorder and $Ba_2Li_xNa_{1-x}Nb_5O_{15}$ solid solution thermodynamics were investigated in this work. The python package bsym [1] was used to determine symmetry inequivalent configurations within the ensemble method, using the formalism outlined in Grau-Crespo et al. [2] 1x1x2 supercells of each $M_2RNb_5O_{15}$ (P4/mbm) composition were generated and fully relaxed using a k-point grid of 2x2x3. Within the ensemble method, Boltzmann probabilities (P_m) are used to determine the existence of a particular unique configuration, m:

$$P_m = \frac{1}{Z}\Omega_m exp\frac{-E_m}{k_B T} = \frac{1}{Z} exp\frac{-E_m}{k_B T} \quad (1)$$

Each of which depends on the energy (E_m) and its associated degeneracy (Ω_m), Z is the partition function, and is related to temperature (T) via the Boltzmann's constant (k_B). The configurational free energy, G, can be obtained thus:

$$G = -k_B T ln(Z) \quad (2)$$

The probability, P_m, can be used to gain a weighted average of an observable quantity (Q) such as the lattice parameters:

$$Q = \sum_{m=1}^{M} P_m Q_m \quad (3)$$

Where M is the total number of inequivalent configurations for a particular configuration.

The tabulated list of configurations and their degeneracy are given below.

Unique Structures and Their Degeneracy

Solid Solutions with A1/A2 Cation Site Disorder

Table S9: A1/A2 cation site disorder in BLNN unique structures and their degeneracy (in brackets).

		x				
		**1**	**0.75**	**0.5**	**0.25**	**0**
	**0**	1 (1)	8 (32)	48 (168)	56 (224)	22 (70)
	**25**	1 (4)	32 (128)	136 (672)	224 (896)	56 (280)
**% offsite**	**50**	3 (6)	48 (192)	222 (1008)	336 (1344)	94 (420)
	**75**	1 (4)	32 (128)	136 (672)	224 (896)	56 (280)
	**100**	1 (1)	8 (32)	48 (168)	56 (224)	22 (70)

Solid Solutions with A1/C Cation Site Disorder

Table S10: A1/C cation site disorder in BLNN unique structures and their degeneracy (in brackets).

		x				
		**1**	**0.75**	**0.5**	**0.25**	**0**
	**0**	1 (1)	8 (32)	48 (168)	56 (224)	22 (70)
	**25**	1 (4)	32 (128)	136 (672)	224 (896)	56 (280)
**% offsite**	**50**	3 (6)	48 (192)	222 (1008)	336 (1344)	94 (420)
	**75**	1 (4)	32 (128)	136 (672)	224 (896)	56 (280)
	**100**	1 (1)	8 (32)	48 (168)	56 (224)	22 (70)

Gibbs Free Energies

Solid Solutions with A1/A2 Cation Site Disorder

Table S11: The Gibbs Free Energies of mixing ΔG_{mix} calculated for A1/A2 cation site disorder within BLNN.

		x				
		**1**	**0.75**	**0.5**	**0.25**	**0**
	**0**	0	-0.0475	-0.06002	-0.03318	0
	**25**	-0.00312	-0.02854	-0.04771	-0.04371	0.02084
**% offsite**	**50**	0.01425	-0.01889	-0.04233	0.015648	0.06981
	**75**	0.032532	0.021903	0.018616	0.087671	0.158803
	**100**	0.0911	0.128757	0.14626	0.205304	0.28759

Solid Solutions with A1/C Cation Site Disorder

Table S12: The Gibbs Free Energies of mixing ΔG_{mix} calculated for A1/C cation site disorder within BLNN.

		x				
		**1**	**0.75**	**0.5**	**0.25**	**0**
	**0**	0	-0.08856	-0.0621	-0.06302	0
	**25**	-0.11999	-0.13935	-0.13121	-0.09405	0.102609
**% offsite**	**50**	-0.15094	-0.1543	-0.12736	0.044469	0.276065
	**75**	-0.14149	-0.12537	0.025045	0.22584	0.462399
	**100**	-0.11209	0.0426	0.228595	0.439767	0.689706

Tabulated Structural Parameters

Solid Solutions with A1/A2 Cation Site Disorder

Table S13: Probability averaged lattice parameters (*a,b,c*, and volume) for A1/A2 cation site disorder inBLNN.

	***a* (Å)**			**x**		
		**1**	**0.75**	**0.5**	**0.25**	**0**
	**0.00**	12.49	12.50	12.55	12.57	12.62
	**25.00**	12.70	12.64	12.67	12.65	12.64
**% offsite**	**50.00**	12.70	12.69	12.71	12.67	12.65
	**75.00**	12.75	12.75	12.76	12.73	12.72
	**100.00**	12.77	12.78	12.77	12.77	12.77
	***b* (Å)**			**x**		
		**1**	**0.75**	**0.5**	**0.25**	**0**
	**0.00**	12.52	12.68	12.61	12.49	12.53
	**25.00**	12.62	12.65	12.67	12.64	12.63
**% offsite**	**50.00**	12.70	12.68	12.71	12.69	12.65
	**75.00**	12.75	12.75	12.76	12.73	12.72
	**100.00**	12.77	12.79	12.80	12.77	12.75
	***c* (Å)**			**x**		
		**1**	**0.75**	**0.5**	**0.25**	**0**
	**0.00**	8.03	7.87	7.87	7.96	7.91
	**25.00**	7.86	7.89	7.89	7.91	7.90
**% offsite**	**50.00**	7.94	7.94	7.91	7.91	7.93
	**75.00**	7.94	7.93	7.91	7.92	7.92
	**100.00**	7.93	7.94	7.93	7.92	7.92
	**Vol. ($Å^3$)**			**x**		
		**1**	**0.75**	**0.5**	**0.25**	**0**
	**0.00**	1256.87	1246.29	1245.29	1249.57	1249.86
	**25.00**	1260.37	1261.40	1267.47	1264.62	1261.19
**% offsite**	**50.00**	1280.49	1277.76	1278.64	1272.53	1267.91
	**75.00**	1289.88	1289.33	1288.15	1284.01	1279.90
	**100.00**	1292.93	1297.30	1295.50	1290.96	1288.96

Solid Solutions with A1/C Cation Site Disorder

Table S14: Probability averaged lattice parameters (*a,b,c*, and volume) for A1/C cation site disorder in BLNN.

		x				
	***a* (Å)**	**1**	**0.75**	**0.5**	**0.25**	**0**
	**0.00**	12.55	12.66	12.62	12.50	12.60
	**25.00**	12.55	12.58	12.55	12.53	12.57
**% offsite**	**50.00**	12.56	12.56	12.54	12.57	12.58
	**75.00**	12.56	12.56	12.58	12.59	12.61
	**100.00**	12.59	12.59	12.59	12.61	12.65
		**x**				
	***b* (Å)**	**1**	**0.75**	**0.5**	**0.25**	**0**
	**0.00**	12.43	12.47	12.58	12.62	12.52
	**25.00**	12.61	12.57	12.58	12.57	12.58
**% offsite**	**50.00**	12.58	12.56	12.56	12.57	12.59
	**75.00**	12.57	12.56	12.58	12.59	12.61
	**100.00**	12.58	12.59	12.60	12.62	12.66
		**x**				
	***c* (Å)**	**1**	**0.75**	**0.5**	**0.25**	**0**
	**0.00**	8.01	7.91	7.84	7.92	7.93
	**25.00**	7.87	7.88	7.91	7.94	7.95
**% offsite**	**50.00**	7.89	7.91	7.93	7.96	7.98
	**75.00**	7.90	7.92	7.94	7.97	7.99
	**100.00**	7.91	7.93	7.97	7.98	7.98
		**x**				
	**Vol. (Å^3)**	**1**	**0.75**	**0.5**	**0.25**	**0**
	**0.00**	1249.99	1247.73	1244.48	1250.14	1250.20
	**25.00**	1244.86	1246.80	1249.05	1250.15	1256.99
**% offsite**	**50.00**	1246.63	1248.35	1249.82	1256.68	1263.24
	**75.00**	1248.11	1250.70	1256.18	1263.37	1270.51
	**100.00**	1251.77	1257.02	1265.07	1270.48	1279.19

References

1. Morgan, B.J., *bsym: A basic symmetry module.* Journal of Open Source Software, 2017. **2**(16): p. 370.
2. Grau-Crespo, R., S. Hamad, C.R.A. Catlow, and N.H. de Leeuw, *Symmetry-adapted configurational modelling of fractional site occupancy in solids.* Journal of Physics-Condensed Matter, 2007. **19**(25).
